# Factors shaping the lichen photobiome and mycobiome in a tropical lichen community

**DOI:** 10.1093/femsec/fiag094

**Published:** 2026-08-14

**Authors:** Magdalena Kosecka, Amélia Bourceret, Benoît Perez-Lamarque, Beata Guzow-Krzemińska, Martin Kukwa, Adam Flakus, Pamela Rodriguez-Flakus, Marc-André Selosse

**Affiliations:** Department of Plant Taxonomy and Nature Conservation, Faculty of Biology, University of Gdańsk, Gdańsk 80-308, Poland; Institute of Ecology and Environmental Sciences of Paris (iEES Paris), Université Paris Est Créteil, CNRS, INRAE, IRD, IEES, F-94010 Créteil, France; Université de Toulouse, Toulouse INP, CNRS, IRD, CRBE, Toulouse 31062, France; Institut de Biologie de l’ENS (IBENS), École normale supérieure, CNRS, INSERM, Université PSL, Paris 75005, France; Department of Plant Taxonomy and Nature Conservation, Faculty of Biology, University of Gdańsk, Gdańsk 80-308, Poland; Department of Plant Taxonomy and Nature Conservation, Faculty of Biology, University of Gdańsk, Gdańsk 80-308, Poland; W. Szafer Institute of Botany, Polish Academy of Sciences, Kraków 31-512, Poland; W. Szafer Institute of Botany, Polish Academy of Sciences, Kraków 31-512, Poland; Department of Plant Taxonomy and Nature Conservation, Faculty of Biology, University of Gdańsk, Gdańsk 80-308, Poland; Institut de Systématique, Évolution, Biodiversité (ISYEB), Muséum national d’Histoire naturelle, CNRS, Sorbonne Université, EPHE, Université des Antilles, Paris 75005, France; Institut Universitaire de France, Paris 75231, France

**Keywords:** lichen symbiosis, holobiont, *Trebouxia* lichen, epiphytic lichens

## Abstract

Lichen thalli host complex microbial communities, defined as a holobiont, which may foster the ecological stability and longevity of the lichen symbiosis. Currently, the understanding of the lichen features that structure the diversity of lichen holobionts in complex lichen communities in natural ecosystems remains incomplete. This study assessed the microbial community diversity and structure in taxonomically diverse co-occurring lichens associated with Trebouxiophyceae algae from Bolivian forests. We focused on three components of the lichen holobiont: the lichenized fungus (mycobiont), its associated algal (photobiome), and fungal (mycobiome) communities. We specifically tested the influence of mycobiont identity, lichen thallus morphological type, reproductive strategy, and secondary metabolites composition on the lichen-associated photobiome and mycobiome. To understand the specialization patterns between holobiont components, we investigated biotic interactions between the mycobiont and the lichen-associated photobiome and mycobiome using network analysis. We observed that co-occurring lichens host diverse, host-specific, yet overlapping photobiomes and mycobiomes. In particular, these microbial communities are influenced by the host’s thallus morphological type and its secondary metabolite content. Finally, we demonstrated that both photobiome and mycobiome are shaped mainly by the mycobiont identity, which results in modular interaction networks, with strong phylogenetic signals and high levels of specialization.

## Teaser

The lichen photobiome and mycobiome are structured primarily by mycobiont identity, resulting in modular networks with strong phylogenetic signals and high levels of specialization.

## Introduction

**Figure 1:**
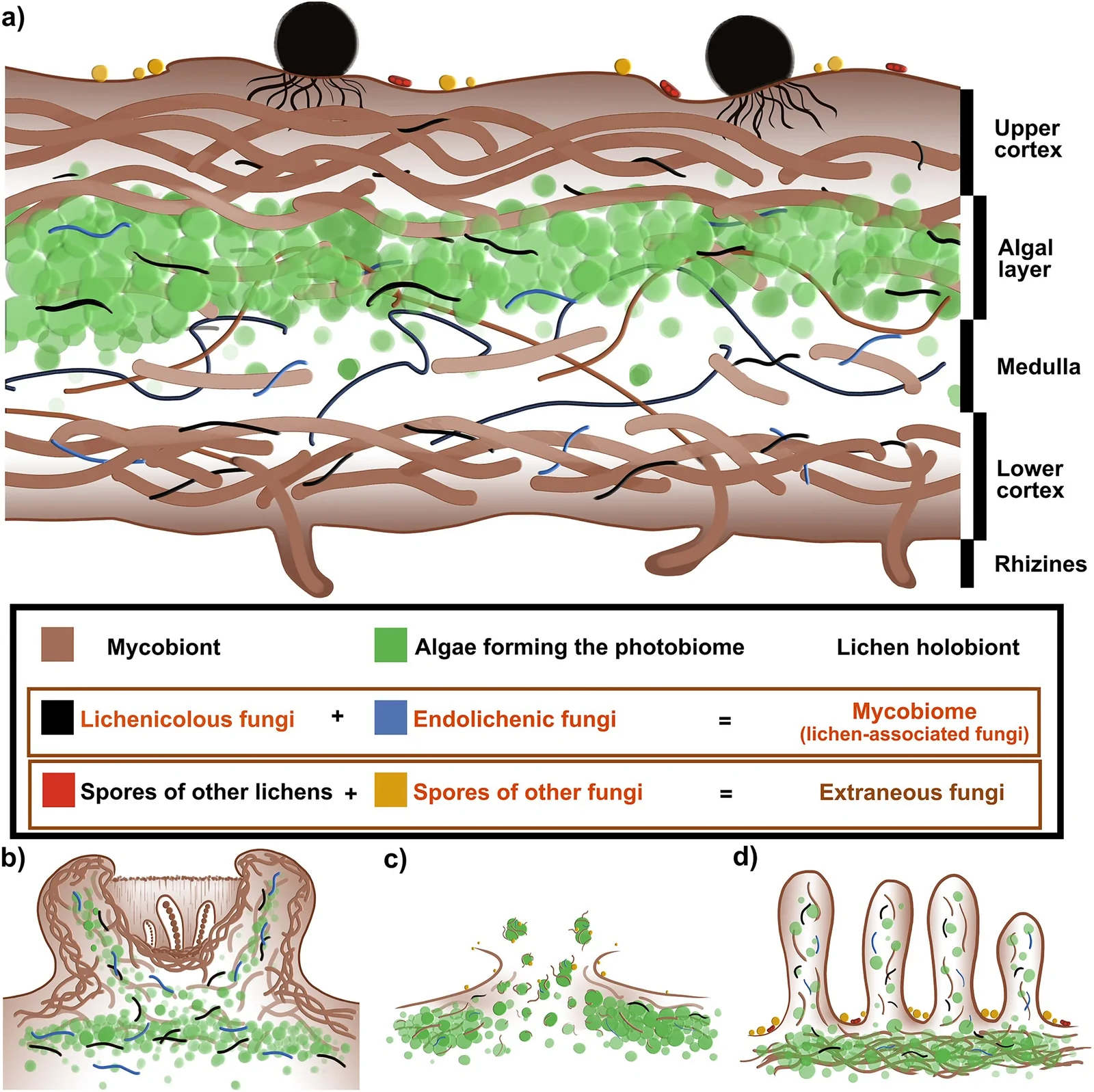
(a) Schematic theoretical cross-section of a lichen thallus with a heteromeric, layered organization. The scheme includes the localization of the lichen-associated fungal community, also called the mycobiome. It is composed of lichenicolous fungi, endolichenic fungi, extraneous fungi that comprise the accidental fungal inhabitants, as well as other lichen spores and vegetative propagules (not shown). The scheme does not present the bacteriome and the virome, which are not the subjects of the current study. (b–d) Schematic theoretical cross-section of selected lichen reproduction structures: fungal apothecium producing ascospores (b), vegetative propagules, soredia produced in soralium (c), and isidia (d). Image by Maciej Pazdro, under a CC BY open access license.

The holobiont concept proposes that a multicellular host and its associated micro-organisms together form a distinct yet dynamic biological entity (Simon et al. 2019). Within this framework, holobiont members collectively maintain functional integrity while exhibiting compositional shifts in response to environmental variation, such as along climatic and chemical gradients (Peay et al. 2017, Rolshausen et al. 2023, Adomako et al. 2025, Glynn et al. 2025). Consequently, the well-being and stability of entire ecosystems, such as coral reefs and forests, depend on the structure and function of complex microbial communities (Vanwonterghem and Webster 2020, Philippot et al. 2021). Lichens represent self-sustaining microecosystems that illustrate the holobiont concept (Hawksworth and Grube 2020, 2024). They play key ecological roles by contributing to primary succession (Brodo et al. 2001), facilitating nutrient cycling (Nash 2008, Elbert et al. 2012), supporting food webs (Purvis 2000), and serving as sensitive indicators of environmental change (Nimis et al. 2002, Markert et al. 2003). Despite their small size, lichens are thus significant drivers of ecosystem functioning. They are composite symbiotic associations between a fungus (the mycobiont, which constitutes the main structural component of the lichen thallus) and photosynthetic partners (photobionts), such as green algae and/or cyanobacteria (Fig. 1a). In addition to these principal partners, lichen thalli harbor diverse associated micro-organisms, including additional algae, fungi, bacteria, and viruses. Collectively, the algal inhabitants constitute the photobiome, while the remaining communities form the mycobiome, bacteriome, and virome, respectively (Spribille et al. 2016, U’Ren et al. 2019, Grube et al. 2015, Ponsero et al. 2021, Hawksworth and Grube 2020), supporting the view of lichens as complex and functionally integrated holobionts.

Understanding the processes shaping the diversity and structure of microbial communities within the lichen holobiont is essential for identifying mechanisms leading to stable lichen symbiosis. However, holobiont assembly—defined as the diversity and organization of all organisms inhabiting the lichen thallus—is governed by a combination of abiotic and biotic factors, resulting in a dynamic equilibrium (Pita et al. 2018). Among abiotic drivers, environmental conditions can influence photobiome composition (Magain and Sérusiaux 2014, Škvorová et al. 2022, Dědková et al. 2025), while both environmental conditions and seasonal climatic variation can alter mycobiome structure and diversity (Beck et al. 2014, Yang et al. 2023). In addition, host ecology and biotic interactions—defined as reciprocal relationships among holobiont components—play a key role in shaping community structure (Bálint et al. 2013, Dal Grande et al. 2018, Glasl et al. 2019).

Host ecology is determined by intrinsic functional traits, including morphological, physiological, and biochemical characteristics that influence microbial associations (Nock et al. 2016, Leiva et al. 2016, Ellis et al. 2021, Mawarda et al. 2025). In lichens, functional traits such as thallus morphology (Wagner et al. 2020, Steinová et al. 2022, Berlinches de Gea et al. 2023), reproductive strategy (Steinová et al. 2019, Černajová and Škaloud 2020, Brunialti et al. 2021), and secondary metabolite production (Lokajová et al. 2014, Khadhri et al. 2019) have been investigated, yet their effects on photobiome and mycobiome structure remain incompletely understood. Thallus, commonly classified as crustose (thin, crust-like, tightly attached to the substrate), foliose (leaf-like, partially attached, more three-dimensional), or fruticose (shrub-like or hair-like, highly branched and erect) based on its morphology, structure, and attachment to the substrate (Nash 2008), may affect microbial diversity. Indeed, the simpler crustose forms, which are tightly appressed to the substrate, have been shown to associate with a broader range of photobionts than the other more structurally complex growth forms (Helms et al. 2001, Wagner et al. 2020, Steinová et al. 2022, Berlinches de Gea et al. 2023).

The reproductive strategy of lichens, combining both asexual and sexual strategies, represents another key shaping factor of microbial assembly within this self-sustaining ecosystem. Sexually reproducing lichens disperse fungal spores that must then acquire photosymbiotic partners from the environment, representing horizontal transmission and potentially increasing algal community diversity (Fig. 1b; Arnold et al. 2009, U’Ren et al. 2010, Dal Grande et al. 2012). In contrast, lichens can preserve the association between the fungal partner (mycobiont) and the photosynthetic partner (photobiont) through asexual reproduction using unique vegetative propagules, such as soredia and isidia. Soredia are small clusters of fungal hyphae and one or a few algal cells (Fig. 1c). Isidia are small outgrowths on the surface or edges of the lichen thallus that contain both fungal and algal cells and are covered by a protective outer layer, resembling the structure of the thallus (Fig. 1d; Nash 2008). This mode of reproduction is referred to as vertical transmission. (Helms et al. 2001, Piercey-Normore 2004, Yahr et al. 2004, Nash 2008, Otálora et al. 2010, Onuţ-Brännström et al. 2017). However, propagule disintegration during germination suggests that partner re-recognition may still be required at this stage, indicating a more complex mechanism than strict vertical inheritance (Černajová and Škaloud 2020).

Despite these differences, both reproductive modes are subject to ecological and evolutionary filtering that determines compatible symbiotic associations (Beck et al. 1998, Yahr et al. 2004, Leavitt et al. 2015, Rodríguez-Arribas et al. 2023). While photobiont dynamics are relatively well characterized (Otálora et al. 2010, Wornik and Grube 2010, Cao et al. 2015, Steinová et al. 2019), the consequences of reproductive strategy for mycobiome assembly remain unknown (Černajová and Škaloud 2020).

Secondary metabolites are a diverse group of organic compounds produced by lichens (mainly the mycobiont) that are not directly involved in primary metabolic processes such as growth or reproduction (Huneck and Yoshimura 1996, Elix and Stocker-Wörgötter 2008). These compounds play important ecological roles, including protection against UV radiation (Legouin et al. 2017, Phinney et al. 2019, Beckett et al. 2021), stress-tolerance (Asplund et al. 2017, Lutsak et al. 2017), allelopathic (Giordano et al. 1997), and antimicrobial properties (Cohen et al. 1996, Ranković and Kosanić 2019), and may therefore regulate both photobiome (Bačkor et al. 1998, 2010, Lokajová et al. 2014) and mycobiome composition (Torzilli et al. 1999, Merinero et al. 2015, Asplund et al. 2018, Khadhri et al. 2019). Additionally, metabolites produced by lichen-associated micro-organisms may contribute to the shape of lichen holobiont community structure (Cernava et al. 2015, Sigurbjörnsdóttir et al. 2015, Spribille et al. 2016, Moreno et al. 2018, Elkhateeb and Daba 2021). However, the overall importance of mycobiont-origin compounds in determining holobiont composition and function remains unclear.

Biotic interactions within holobionts can be evaluated using network-based approaches, which describe community organization through metrics such as connectance, nestedness, and modularity (Barberán et al. 2012). Connectance refers to the proportion of possible interactions that are actually realized within the network, reflecting how densely connected the community is (Newman 2010). Nestedness describes a pattern in which the interactions of specialists are subsets of those of more generalist species, indicating a hierarchical structure of interactions (Bascompte et al. 2003). Modularity measures the extent to which the network is divided into relatively independent groups of species that interact more strongly with each other than with the rest of the community (Olesen et al. 2007). In lichens, host specialization toward specific photobionts and fungal associates has been widely studied, particularly in the photobiome (Kaasalainen et al. 2021, Berlinches de Gea et al. 2023, Blázquez et al. 2025), and in the mycobiome (De Carolis et al. 2023, Yang et al. 2023). Across these studies, a consistent pattern emerges that lichens are structured by non-random, often host-associated selection of photobiome and mycobiome, but the strength and specificity of this selection vary between symbiont groups. However, broader ecological implications of network properties (Chagnon et al. 2018, Duran-Nebreda and Valverde 2023, Pérez-Ortega et al. 2023) still need to be further explored to better understand biotic interactions within the lichen holobiont.

The mycobiome, corresponding to the fungal community associated with lichen, can be divided into three functional groups (Fernández-Mendoza et al. 2017, Fig. 1a): lichenicolous fungi, endolichenic fungi (residing inside the thalli), and extraneous fungi (spores and vegetative propagules that land on the thallus). Despite increasing interest in lichen-associated microbiota, relatively few studies have examined the relationship between photobiome and mycobiome diversity in natural systems (Mark et al. 2020, Xu et al. 2022). Interactions between these microbial communities may play an important role in shaping holobiont structure and its functioning. Endolichenic fungi are frequently located within the algal layer, suggesting potential ecological and evolutionary interactions with photobionts (Arnold et al. 2009). Experimental studies have revealed close physical associations between these inhibiting lichen organisms, including direct cell-to-cell contact and the formation of specialized interaction structures, like mucilage production at interaction zones, and occasionally the formation of haustorium-like structures (Gorbushina et al. 2005, Muggia et al. 2016). These observations indicate that nutrient exchange and other direct interactions between photobionts and associated fungi may contribute to shaping mycobiome composition. Together, this evidence supports the hypothesis that variation in mycobiome structure may be influenced not only by the identity of the mycobiont but also by the composition of the photobiome (Muggia et al. 2016).

Next-generation sequencing of algal and fungal communities inhabiting lichen enables comprehensive characterization of lichen-associated photobiome and mycobiome without the need for cultivation. Comparing co-occurring lichen species—i.e. species sharing the same habitat—that differ in functional traits provides an opportunity to disentangle the intrinsic and extrinsic factors shaping these communities. We hypothesize that, in addition to lichen functional traits, environmental conditions and host mycobiont specialization govern the diversity and structure of the lichen photobiome and mycobiome. To test this, we sampled different mycobiont (lichenized fungi) species with Trebouxiophyceae photobionts across five Neotropical forest sites. Specifically, we aimed to: (i) assess the influence of mycobiont identity and functional traits on photobiome and mycobiome diversity and community structure; (ii) reconstruct interaction networks among holobiont components (mycobiont–photobiome, mycobiont–mycobiome, and photobiome–mycobiome) and quantify their structure and specialization; and (iii) evaluate the effects of functional traits and environmental conditions on interaction network properties.

## Experimental procedures

### Sampling strategy

The study was conducted in two Neotropical forest regions in Bolivia, encompassing five sampling sites with diverse epiphytic lichen assemblages. The selected forests host lichen-forming fungi with different thallus morphologies (crustose, foliose, and fruticose), and various reproductive strategies (apothecia, vegetative propagules, or both), producing a variety of secondary metabolites (∼43). Most dominant lichen species in these forests associate with green algal photobionts belonging to the class Trebouxiophyceae, and these lichens are classified particularly in the families Parmeliaceae and Physciaceae. In contrast, lichens associated with photobionts from the Trentepohliaceae (e.g. Arthoniaceae and Graphidaceae) or with dual symbioses involving green algae and cyanobacteria (e.g. Lobariaceae) were comparatively rare or had thalli too small for standard sampling. To reduce variation arising from fundamentally different symbiotic systems, the sampling focused exclusively on the dominant epiphytic lichen-forming fungal taxa associating with Trebouxiophyceae photobionts.

Field sampling was carried out in November 2016 in two regions of the northern Yungas Forest in Bolivia: (1) Carrasco National Park, Department of Cochabamba, and (2) the Department of La Paz. Within these regions, five sites of ∼1 km² were selected at comparable elevations but differing in levels of anthropogenic disturbance. Sampling focused on epiphytic lichens growing on the dominant tree species, *Alnus acuminata*, to reduce variation related to substrate type.

Three sites were located within Carrasco National Park (CB1–CB3; Table 1), representing a mountain cloud forest ecosystem. Two nearby sites, CB1 and CB2 (∼400 m apart), were selected for intensive community-level sampling. Site CB1 was affected by anthropogenic disturbance, including cattle grazing and selective logging, whereas CB2 showed minimal human impact and no visible signs of grazing or logging. At each site, 15 individuals of *A. acuminata* were selected. From each tree, 12 lichen specimens representing the local species diversity were collected at heights of 130–180 cm above ground, resulting in 360 specimens in total. An additional site (CB3) was included to increase regional biodiversity coverage; at this site, 28 additional lichen specimens were collected from *A. acuminata*.

**Table 1:** Sampling localities description.

Locality designation	Number of samples	Locality information	GPS information	Elevation	Anthropogenic status	Habitat type
CB1	180	Department Cochabamba, Parque Nacional Carrasco, Province Carrasco	17°29′57″S, 65°16′30″W	2235 m a.s.l	disturbed	mountain cloud forest
CB2	180	Department Cochabamba, Parque Nacional Carrasco, Province Carrasco	17°29′43″S, 65°16′29″W	2210 m a.s.l	minimally influenced	mountain cloud forest
CB3	28	Department Cochabamba, Parque Nacional Carrasco, Province Carrasco	17°33′24″S, 65°16′10″W	2760 m a.s.l	minimally influenced	mountain cloud forest
LP1	53	Department La Paz, Province Franz Tamayo	14°40′31″S, 68°25′05″W	1500 m a.s.l	minimally influenced	savanna
LP2	35	Department La Paz, Province Franz Tamayo	14°38′51″S, 68°24′44″W	1520 m a.s.l	disturbed	savanna

Two sites were sampled in the Department of La Paz (LP1–LP2; Table 1), representing a more open (with fewer trees), savanna-like ecosystem. These sites were included to assess lichen, photobiome, and mycobiome diversity across a broader regional context and to contrast patterns observed in Carrasco National Park. Site LP1 showed relatively low levels of disturbance (minor grazing and logging), whereas LP2 was more strongly affected by anthropogenic activity. The two sites were located at similar elevations (around 1500 m) and separated by ∼2.8 km. From *A. acuminata* trees, 53 and 35 lichen specimens were collected at LP1 and LP2, respectively.

Overall, this hierarchical sampling design—spanning multiple host species, functional traits (thallus morphology, reproductive strategy, and secondary metabolite production), trees, sites, and disturbance levels—enabled the assessment of how mycobiont identity, environmental conditions, and host traits influence the diversity and structure of associated photobiome and mycobiome communities within the lichen holobiont.

All specimens were air-dried after field sampling and frozen at −20°C until further procedures.

Lichen specimens have been collected and deposited in the lichen collections of the University of Gdańsk (UGDA), Institute of Botany of the Polish Academy of Sciences (KRAM), Herbario Nacional de Bolivia, Universidad Mayor de San Andrés, and Museo Nacional de Historia Natural La Paz (LPB) in 2016. The specimens have been collected in accordance with all rules regarding protected areas after receiving the relevant permissions. Material collection has been performed in cooperation with Universidad Mayor de San Andrés, La Paz.

### Distribution of lichen species within sampling sites and similarity analyses

To assess differences in lichen species composition between sampling sites, particularly between the intensively sampled Carrasco sites (CB1 and CB2) and the La Paz sites (LP1 and LP2), both presence–absence and abundance-based similarity indices were applied.

Species composition similarity based on presence/absence data was calculated using the Jaccard index (Jaccard 1901):


\begin{eqnarray*}
J = \frac{a}{{a + b + c}},
\end{eqnarray*}

where *a* represents the number of shared species between two sites (e.g. CB1 ∩ CB2, LP1 ∩ LP2), *b* the number of species unique to the first site, and *c* the number of species unique to the second site used in comparison.

To account for differences in species abundances, the Bray–Curtis similarity index (Bray and Curtis 1957) was used:


\begin{eqnarray*}
BC = \frac{{2C}}{{{N}_1 + {N}_2}},
\end{eqnarray*}

where *C* is the sum of shared species specimens (minimum abundances across sites), and *N₁* and *N₂* represent the total number of specimens in the first and second site, respectively.

### Taxonomic identification and functional trait assessment

Lichen specimens were identified to species level based on thallus morphology, anatomy, and secondary metabolite profiles. Secondary metabolites were analysed using thin-layer chromatography (TLC) following Culberson and Kristinsson (1970), and Orange et al. (2001). Compounds were identified to the finest possible level and assigned to substance classes according to Elix (2014). To evaluate the influence of secondary metabolites on photobiome and mycobiome structure, identified compounds were grouped into 12 substance classes. For each specimen, metabolite classes were coded as present (1) or absent (0). A complete overview of specimen metadata is provided in Supplementary Table S1.

### DNA isolation, PCR, and DNA metabarcoding

A small piece of thallus (between 2 and 5 mg of dry mass) was used without surface sterilization from each lichen specimen to characterize the lichen photobiome and mycobiome. Priority was given to thalli fragments with a visible presence of lichenicolous fungi to include them within the mycobiome characterization. Total genomic DNA of lichens was extracted using the Plant & Fungi DNA Purification Kit (Eurx, Poland), following the manufacturer’s instructions. The concentration of each DNA sample was measured by fluorescence (Quant-iT PicoGreen; Invitrogen, USA) and then diluted to a final concentration of 3.5 ng/μl.

To assess the overall diversity of the lichen photobiome and mycobiome, we used metabarcoding. To profile fungal communities, comprising the mycobiont and lichen-associated mycobiome we amplified ITS2 rDNA region using ITS86-F (Turenne et al. 1999), and ITS4 primers (White et al. 1990), while to profile algal communities that form the photobiome, comprising the photobionts as well as possible epiphytic algae invisible to the bare eye, we amplified ITS2 rDNA region, using FDGITS2-f and FDGITS2-r primers (Dal Grande et al. 2018).

Polymerase chain reaction was performed with 25 µl of reaction mixture, containing 0.2 mM dNTPs, 0.3 mM of each primer, 2 units of DFS-Taq DNA Polymerase (BIORON, Germany), 1× Incomplete Buffer supplied with MgCl_2_, BSA 3%, and 3 µl of template DNA. In the case of fungal communities amplification, the following program was used: initial denaturation, 10 min at 95°C; 30 cycles of: denaturation, 30 s at 95°C; annealing, 30 s at 56.5°C; and elongation, 30 s at 72°C, followed by final elongation, 7 min at 72°C. Amplification of algal communities was performed with the following cycle conditions: initial denaturation, 4 min at 95°C; 35 cycles of denaturation, 30 s at 95°C; annealing, 20 s at 54°C; and elongation, 20 s at 72°C, followed by final elongation, 5 min at 72°C.

Each sample was amplified in three independent PCR replicates (technical replicates) to reduce amplification bias. A unique pair of barcoded primers was assigned to each sample, and the triplicate PCR products were pooled prior to library preparation and sequencing. Each PCR plate included a control replicate without a DNA template and two additional negative controls (ultrapure water), resulting in eight negative controls per library. PCR products were evaluated on a 1% agarose gel with Midori Green staining, purified with Ampure XP beads (Agencourt, Beckman Coulter, USA), quantified by fluorescence, and pooled in equimolar amounts to construct a single library per group (fungi or algae). Each library was purified twice and quantified prior to sequencing. Sequencing was performed on an Illumina MiSeq platform (paired-end 2 × 250 bp) at Fasteris, Switzerland. Library preparation and sequencing followed an adapted protocol from Bourceret et al. (2022) using a multiplexing approach, as described in Petrolli et al. (2021).

### Amplicon sequencing data processing

Sequencing outputs from the two libraries (the ‘fungal dataset’ and ‘algal dataset’) were separately processed using VSEARCH (Rognes et al. 2016) following Perez-Lamarque et al. (2022a). In short, paired-end reads were assembled, quality-checked, and demultiplexed with cutadapt (Martin 2011). Fungal reads were clustered into 97% operational taxonomic units (OTUs), while algal reads were processed into amplicon sequence variants (ASVs) following the recommendations of Blázquez et al. (2022). Clustering algal reads into 97% OTUs provided qualitatively similar results. Therefore, only results on ASVs are reported here for algae. Fungal OTUs and algal ASVs were checked for chimeras. For fungal taxonomic assignments, we used UNITE (Nilsson 2019), and enriched the database with reference sequences of fungal mycobionts obtained using Sanger sequencing (produced for the study of Kosecka et al. 2022, not published therein; Methods S1, Table S1.1). For algae, we used Silva (Quast et al. 2013), amended with *Trebouxia* sequences from the study of Kosecka et al. (2022). Both databases were customized as described above and further trimmed to the target amplicon region based on the primer sequences. Taxonomic assignments were performed on non-chimeric fungal OTUs and algal ASVs using VSEARCH v2.15.0 (Rognes et al. 2016). Representative sequences were compared against the corresponding curated reference database using the usearch global algorithm, which performs global pairwise sequence alignments. For each query sequence, only the best-matching reference sequence was retained, and taxonomic assignments were based on sequence similarity. Searches were conducted with a minimum identity threshold of 50%, allowing up to 32 rejected matches and no limit on accepted matches. Contaminants identified in the negative controls were filtered out of the OTU/ASV tables using the decontam pipeline (Davis et al. 2018). We removed OTU/ASV present by less than five reads and converted the abundances into relative abundances while only retaining OTU/ASV representing at least 1% of the reads in each sample (Perez-Lamarque et al. 2023). The few samples with fewer than 1000 algal reads or less than 100 mycobiome reads were discarded in the following analyses. The following analyses were performed in R (R Core Team 2023) using vegan and Plyr packages (Oksanen et al. 2012, Wickham 2011). The script description and the output files, along with links to them, are available in Methods S2. We prepared three different OTU/ASV tables that described different components of the lichen holobiont: the mycobiont, the photobiome, and the mycobiome [including all lichen-associated fungi: lichenicolous, endolichenic, and extraneous fungi, but excluding the mycobiont(s)].

For the fungal dataset of (i) the mycobiont OTUs, we extracted the OTUs corresponding to the main lichenized fungi from each sample. Next, we checked that the taxonomic assignments of the mycobiont identified using metabarcoding matched those based on lichen thallus morphology, anatomy, and secondary metabolite profiles. We only kept samples with matching taxonomic assignments (Table S1).

In the case of (ii), the mycobiome OTUs (non-lichenized fungi), we kept a complete dataset that passed the above filtering procedure (OTU > 5 reads, OTU with a minimum of 5% reads per sample). To identify the trophic category for each mycobiome’s OTU, we first used FUNGuild (Nguyen et al. 2016) to assign a guild, i.e. pathotroph, symbiotroph, or saprotroph. This was considered the first rough assignment of trophic mode to fungi in the lichen mycobiome, validated after expert visual checking (e.g. removing animal or plant pathotrophs from extraneous fungi). This approach does not offer the exactness of the delineation of lichenicolous, endolichenic, and extraneous fungi, which may contain OTUs that we attribute to all guilds. However, the exact delimitation of the location and role of a particular fungus is beyond the scope of the present research.

Next, we reconstructed a phylogenetic tree of (i) all the mycobiont OTUs (lichenized fungi), (ii) all mycobiome OTUs (non-lichenized fungi), and (iii) all algal ASVs following Perez-Lamarque et al. (2022b, 2023). Sequences were aligned using MAFFT (v7; Katoh and Standley 2013) with the auto strategy, which selects the most appropriate alignment algorithm based on dataset size and sequence divergence. Ambiguously aligned regions were removed using trimAl (v1.4; Capella-Gutiérrez et al. 2009), applying the automated heuristic mode, which filters alignment columns based on gap frequency and local alignment reliability criteria. Multiple sequence alignments were then analysed in IQ-TREE (v2.1.1; Nguyen et al. 2015), using ModelFinder (Kalyaanamoorthy et al. 2017) for model selection under the Bayesian Information Criterion (BIC; Burnham and Anderson, 2002). The sequence alignment characteristics and the best-fitting nucleotide substitution models for each dataset (i–iii) are summarized in Table S2. Maximum-likelihood phylogenetic trees were inferred under the selected models, and branch support was assessed using 1000 SH-aLRT and 1000 ultrafast bootstrap replicates. Phylogenetic trees based on the ITS marker usually present a significant level of uncertainty. Yet, they can still be informative for ecological alpha and beta diversity analyses, serving as a complement to non-phylogenetic metrics (Perez-Lamarque et al. 2022c).

We proceeded with further analysis to respond to our three objectives, using the different variants of prepared datasets: (i) mycobiont OTUs (lichenized fungi), (ii) mycobiome OTUs (non-lichenized fungi), and (iii) algal ASVs; either using all sampling sites, or only CB1 and CB2.

### Diversity and community composition analyses

To assess mycobiont, photobiome, and mycobiome diversity across a broader regional context, and to incorporate environmental variability across regions and disturbance level (Objective 1), diversity and community composition analyses were performed using the full dataset. These analyses incorporated (i) mycobiont identity at the order and genus levels, (ii) thallus morphological type, (iii) lichen reproductive strategy, and (iv) sampling site.

Sampling completeness was evaluated using sample-based rarefaction analyses implemented in R. For each site, OTU/ASV tables were converted to presence–absence data and randomly subsampled without replacement across increasing numbers of samples. For each sampling level, 100 randomizations were performed, and the mean observed OTU/ASV richness was calculated. In addition, Faith’s phylogenetic diversity (PD) was estimated for each subsample using the *pd* function in the R package picante (Kembel et al. 2010). Rarefaction curves were generated by plotting mean richness and phylogenetic diversity against the number of samples collected at each site.

Rarefaction analyses of observed OTU/ASV richness indicated that sites CB3, LP1, and LP2 were far from reaching saturation, whereas sites CB1 and CB2 approached a plateau (Fig. S1), consistent with the sampling depth at each site. Similar patterns were observed for Faith’s phylogenetic diversity (PD), with CB1 and CB2 approaching saturation while the remaining sites showed evidence of incomplete sampling (Fig. S1). Therefore, analyses aimed to evaluate correlations between secondary metabolites and community composition (Objective 1), and biotic interactions, including interaction networks, their structure and specialization patterns (Objectives 2, 3), were restricted to samples from sites CB1 and CB2. This restriction was applied to reduce environmental heterogeneity and potential confounding effects. It was further supported by the more complete sampling coverage at these sites, thereby enabling more robust inference of biotic interactions within a more controlled subset of communities. To address our first objective—determining the influence of mycobiont identity and selected functional traits on photobiome and mycobiome community diversity and structure—we analyzed patterns of community composition based on the OTU/ASV tables generated for both microbial datasets. Specifically, we examined whether variation in photobiome and mycobiome composition was associated with four explanatory factors: (i) mycobiont identity at the order and genus levels; (ii) thallus morphological type; (iii) lichen reproductive strategy; and (iv) sampling site (Table S1). Variation in the photobiome and mycobiome was assessed using both alpha and beta diversity metrics.

Beta diversity was calculated using the Bray–Curtis dissimilarity index implemented in the R package vegan (Oksanen et al. 2012). Patterns of community similarity were visualized using principal coordinate analysis (PCoA) based on Bray–Curtis distances, performed with the *cmdscale* function in the R base package (R Core Team 2023). To test whether community composition differed significantly among the defined groups of the four explanatory factors, we conducted distance-based redundancy analysis (dbRDA) and Permutational Multivariate Analysis of Variance (PERMANOVA) implemented with *capscale, anova.cca*, and *adonis2* in the vegan R package (Oksanen et al. 2012). PERMANOVA significance was assessed using 999 permutations. These analyses allowed us to evaluate the relative influence of mycobiont identity, host functional traits, and site conditions on the structure of the lichen-associated photobiome and mycobiome. Although PERMANOVA is relatively robust to heterogeneity of dispersions in balanced designs (Anderson and Walsh 2013), we tested the assumption of homogeneity of multivariate dispersions using a permutation-based procedure (PERMDISP) implemented with the *betadisper* function in the vegan R package (Oksanen et al. 2012). This analysis was performed using the same distance metric as in the PERMANOVA, with 1000 permutations. In addition, to evaluate the robustness of our results with respect to sampling design, we repeated the PERMANOVA analyses using only the two deeper sampled sites (CB1 and CB2).

To further address the first objective, we assessed differences in alpha diversity of photobiome and mycobiome communities. Alpha diversity was calculated for each lichen sample from the five sampling sites using observed OTU/ASV richness and the Shannon index (function *diversity* in the R package vegan; Oksanen et al. 2012). Differences in alpha diversity were tested according to the same four explanatory factors (mycobiont identity at the order and genus level, thallus morphology, lichen reproductive strategy, and sampling site), using Kruskal-Wallis tests.

In the case of secondary metabolites, we used a correlation-based approach to assess the relationship between the presence of particular secondary metabolites in the lichen thalli and the community composition of the lichen photobiome and mycobiome. We tested whether lichens with similar secondary metabolites tend to host similar photobiomes or mycobiomes using Mantel tests (Mantel 1967; function *mantel* in the ecodist R package; Goslee and Urban 2007). For each site (CB1 and CB2), for each component of the lichen holobiont (photobiome, mycobiome), beta-diversity between samples was analysed using weighted UniFrac distances (Lozupone and Knight 2005), while distances for secondary metabolites were computed using binary Jaccard distances (Jaccard 1901, Chung et al. 2019). The strength of the relationships was assessed using Spearman correlation coefficients, with significance evaluated based on 10 000 permutations. Furthermore, we tested whether lichens with particular secondary metabolites tend to host richer photobiome or mycobiome communities (including fungal trophic modes). The significance of the results was checked by using the Wilcoxon test.

### Reconstruction and analysis of interaction networks

To address our second and third objectives, based on the OTU/ASV tables from sites CB1 and CB2, we reconstructed three types of bipartite interaction networks per site: (i) mycobiont–photobiome, (ii) mycobiont–mycobiome, and (iii) photobiome–mycobiome. We built quantified OTU/ASV-level networks; for instance, for the mycobiont–photobiome network, we counted the number of samples in which each pair of mycobiont OTU and algal ASV co-occurred. We plotted the networks using the Fruchterman–Reingold layout algorithm (Fruchterman and Reingold 1991) implemented in the igraph R package (Csardi and Nepusz 2006), which extracts the position of the nodes that reflects the similarity in species interactions. For the purpose of the visualization, we reconstructed the bipartite interaction network of mycobiont–photobiome using the data from all the samples (N = 309).

We evaluated the structure of each network in R following Perez-Lamarque et al. (2023), using several established metrics. First, we computed the connectance as the ratio of observed interactions relative to all possible interactions (Dormann et al. 2008). Second, the nestedness—the tendency of specialist taxa to interact with generalist taxa, which forms an interaction core—was quantified using the *nested* function with the weighted NODF method (Almeida-Neto et al. 2008). Third, modularity, representing the tendency of subsets of taxa to interact preferentially with one another, was computed using Beckett’s algorithm in the *computeModules* function (Dormann et al. 2008, Beckett 2016). Fourth, the network-level H2’ index of interaction specialization (*H2fun* function; Blüthgen et al. 2006) was calculated to assess whether interactions were random (H2’ ≈ 0) or highly specialized (H2’ ≈ 1) (Dormann et al. 2008). We assessed the influence of lichen functional traits (morphological type and reproductive strategy) and site conditions on the H2’ index across different datasets.

To assess the statistical significance of network indices (connectance, nestedness, modularity, and H2’), we compared observed values to null distributions generated by randomizing OTUs/ASVs tables. For this purpose, sample names were shuffled 100 times independently; for each shuffled table, networks were reconstructed, and indices recalculated. The 95% confidence intervals of the null distributions were used to determine significance; observed values falling outside these intervals were considered statistically significant. This procedure ensured that observed network patterns were not artifacts arising from sample aggregation during network reconstruction.

To assess whether network structure was sensitive to the taxonomic resolution used for the photobiome, we repeated all mycobiont–photobiome network analyses using algal OTUs instead of ASVs. Specifically, connectance, nestedness, modularity, and network-level specialization (H2′) were recalculated from OTU-based networks and compared with the corresponding ASV-based results. Because both approaches yielded qualitatively identical patterns, including the relative differences among sites and overall network structure, the main analyses are presented using algal ASVs, which provide finer taxonomic resolution.

Furthermore, phylogenetic signals in interactions within each network were quantified for each component (mycobiont, photobiome, mycobiome) using Mantel tests (Mantel 1967, Perez-Lamarque et al. 2022c). For each component, we compared (i) a phylogenetic distance matrix among taxa with (ii) a dissimilarity matrix describing differences in their interaction partner composition. Interaction-based dissimilarity matrices were constructed by representing each taxon (e.g. each mycobiont OTU, algal ASV, or fungal OTU) by the identity and relative frequencies of its interactions within the bipartite networks (e.g. algal ASVs or fungal OTUs associated with each mycobiont OTU). Pairwise dissimilarities among taxa were then calculated using Bray–Curtis (Bray and Curtis 1957) or UniFrac distances (Lozupone and Knight 2005). Spearman correlations between phylogenetic and interaction dissimilarity matrices were computed, and significance was assessed using 10 000 permutations.

To address our third objective, we assessed the influence of the selected lichen functional traits (morphological type and reproduction strategy), and site conditions on the H2’ index. This analysis was conducted on bipartite networks reconstructed from subsets of the data, grouped according to these functional traits. To assess the influence of each holobiont component identity, we also examined the specialization of individual OTUs/ASVs using the Net Relatedness Index (NRI), which quantifies the mean pairwise phylogenetic distance among all interaction partners of a given taxon. Positive NRI values indicate that a taxon tends to specialize toward closely related partners (Webb et al. 2002). We reported NRI values for mycobiont genera, algal species, and mycobiome-forming fungal orders or ecological guilds inferred using FUNGuild (pathotrophs, symbiotrophs, saprotrophs). The effects of taxonomic groups and ecological guilds on NRI values were tested using the Kruskal–Wallis test.

## Results

### Lichen species distribution and similarity among sampling sites

The comparison between CB1 and CB2 revealed moderate similarity in species composition (*J* = 0.29), with 23 shared species, while abundance-based similarity was higher (*BC* = 0.56), indicating comparable dominance structures despite compositional differences. In contrast, the LP1–LP2 comparison showed low similarity both in species composition (*J* = 0.09; 3 shared species) and abundance (*BC* = 0.14), reflecting stronger differentiation between these savanna sites. A summary of species richness, shared taxa, and similarity indices is provided in Table 2. CB3 was excluded from similarity analyses due to its low sampling intensity (fewer than 10 specimens representing six species), which could bias comparative metrics.

**Table 2:** Summary of lichen species richness, number of shared and unique species, total number of specimens, and similarity indices between sampling sites.

Site	Mycobiont species	Shared species	Unique species	Jaccard index [*J*]	Specimens	Shared-species specimens	Bray–Curtis similarity index [*BC*]
CB1	55	23	32	0.29	132	66	0.56
CB2	47		24		120	75	
LP1	24	3	21	0.09	29	3	0.14
LP2	12		9		12	3	

### The photobiome and mycobiome composition

From the 348 lichen specimens that were successfully amplified for both the photobiome, and the mycobiome, 309 samples were kept after filtering (Table S1). Within these, we identified 111 mycobiont OTUs, 183 algal ASVs (corresponding to 156 OTUs; ASVs provide finer resolution and are therefore reported here), and 1100 non-lichenized fungal OTUs representing the mycobiome (raw data summary is provided in Table S3).

**Figure 2:**
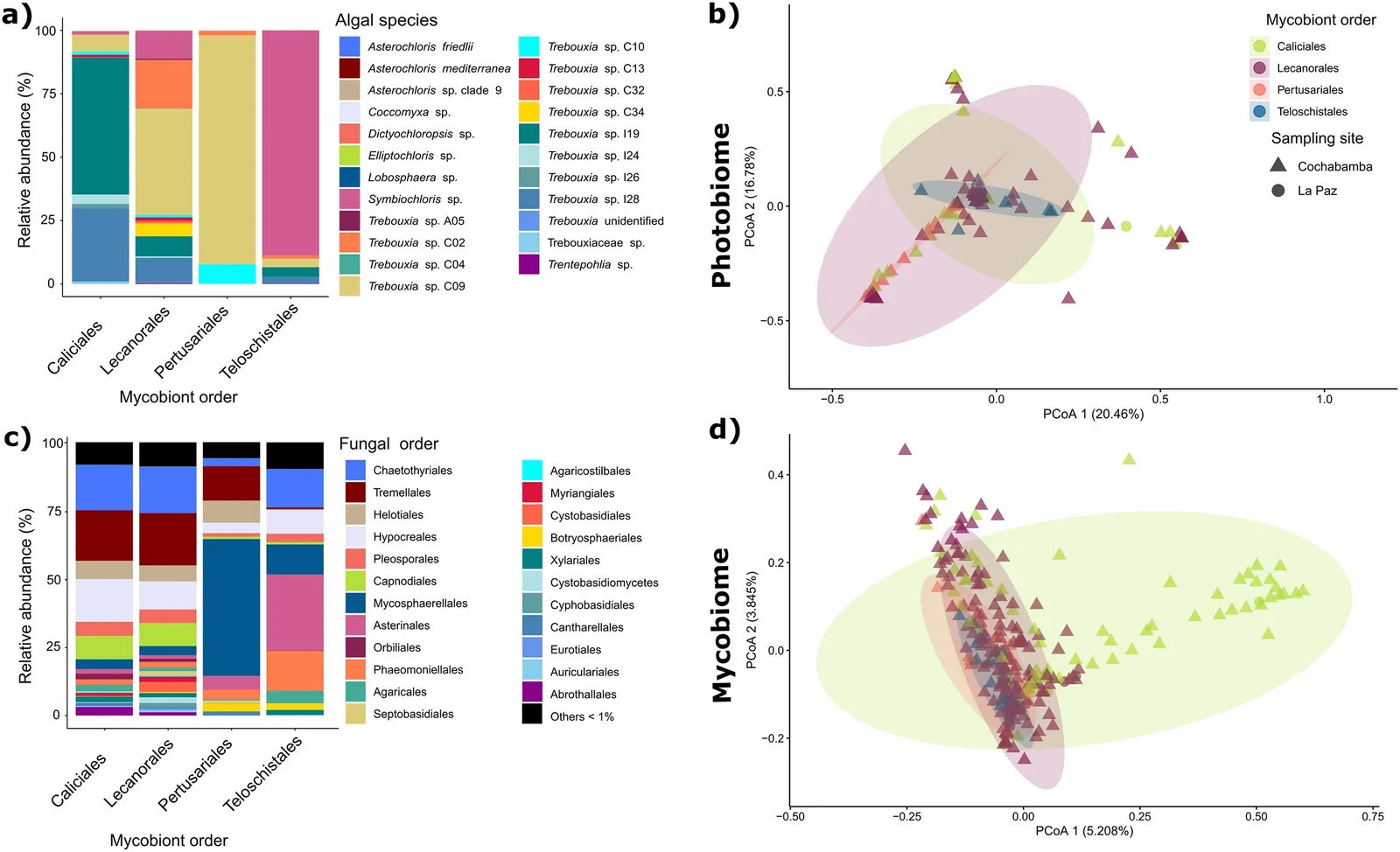
Photobiome and mycobiome composition across lichen specimens (n = 309) at the order level. Relative abundances of the most abundant algal (a) and fungal (c) taxonomic groups are shown at the species level (photobiome) and order level (mycobiome), respectively. Only the main taxonomic groups representing the majority of reads are displayed; remaining taxa were omitted to complete the total to 100%. For algae (a), the legend is organized alphabetically, while for fungi (c), the legend is ordered from the most abundant to the least abundant fungal orders. Principal Coordinates Analysis (PCoA) based on Bray–Curtis dissimilarity was performed for all specimens (b, d). Points’ shapes indicate the sampling site (Cochabamba or La Paz), and their colors represent mycobiont identities at the order level. The shaded polygons delineate mycobiont identities at the order level. The dbRDA and PERMANOVA analyses revealed significant differences among mycobiont orders in both photobiome (dbRDA explained 10.8% of the variation, *P* = 0.001; PERMANOVA: R² = 0.11, *P* = 0.001) and mycobiome structure (dbRDA explained 4.56% of the variation R² = 0.05, *P* = 0.001).

The photobiome was dominated by *Trebouxia*, which accounted for the majority of detected algal ASVs across samples (Fig. 2a), except for Teloschistales mycobionts, whose photobiome is mainly formed by *Symbiochloris* (another genus within Trebouxiophyceae; Fig. 2a, Table S4a). Equivalent analyses performed at the algal OTU level yielded the same qualitative patterns of community composition and taxonomic structuring observed for ASVs (Fig. S3), supporting the robustness of our conclusions to the choice of taxonomic resolution. Mycobiome composition was broadly similar among lichen species belonging to Caliciales and Lecanorales. In contrast, the lichen species in the orders Pertusariales and Teloschistales have mycobiomes dominated by Mycosphaerellales and Asterinales, respectively (Fig. 2c, Table S4b).

### Factors shaping the photobiome and mycobiome composition

First, we tested the assumption of homogeneity of multivariate dispersions using a permutation-based approach (PERMDISP). For the photobiome, dispersions did not differ significantly among sites (*P* = 0.072; Table S5), indicating that the assumption of homogeneous variance was fulfilled and supporting the robustness of the PERMANOVA results. In contrast, the mycobiome showed significant heterogeneity of dispersions among sites (*P* = 0.001; Table S5). However, pairwise comparisons revealed that this effect was not consistent across all sites, but was driven by a limited number of site pairs, for LP2–CB1, CB3–CB2, and LP2–CB2, while the rest of the site pairs were not significant. This suggests that dispersion heterogeneity is present but not pervasive across the entire dataset. Therefore, we performed the diversity and community composition analyses using the full dataset (Tables S6a, S7a) and separately (Tables S6b, S7b) for samples from sites CB1 and CB2.

#### The mycobiont identity

The mycobiont identity, at the order and genus levels, is the primary source of variation in photobiome and mycobiome community composition (Fig. 2 and S2b, S2d). Moreover, it is the leading source of variation in relative abundance for all fungal trophic modes within the mycobiome, i.e. pathotrophs, symbiotrophs, and saprotrophs (Fig. S4). PCoA revealed clustering patterns in both photobiome (Fig. 2b) and mycobiome (Fig. 2d) community composition according to mycobiont taxonomic assignment (order level), providing a visual representation of the community structure. The mycobiomes were slightly clustered on the first axis (5.2% of variance) according to the mycobiont orders Lecanorales, Pertusariales, and Teloschistales, whereas samples assigned to the mycobiont order Caliciales were more dispersed (Fig. 2d). Similarly, we observed a slight clustering of samples according to the mycobiont genus (Fig. S2c; e.g. *Hypotrachyna, Usnea*, and *Parmotrema*). This clustering pattern was weaker for the photobiome, indicating lower taxonomic structuring compared to the mycobiome at both levels: mycobiont order (Fig. 2b) and genus level (Fig. S2a). The dbRDA and PERMANOVA analyses further confirmed a significantly stronger effect of the mycobiont identity at the genus level on the photobiome and mycobiome community structure. In the dbRDA models, mycobiont genus explained 25.46% and 14.71% of the variation in photobiome and mycobiome composition, respectively (Table S6a), whereas mycobiont order explained 10.8% and 4.56% of the variation (Table S6a). These patterns were consistent with the PERMANOVA results (Table S7a; R² = 0.26 and 0.15 for genus level, and R² = 0.11 and 0.05 for order level, respectively).

#### The morphological type of lichen thallus

**Figure 3:**
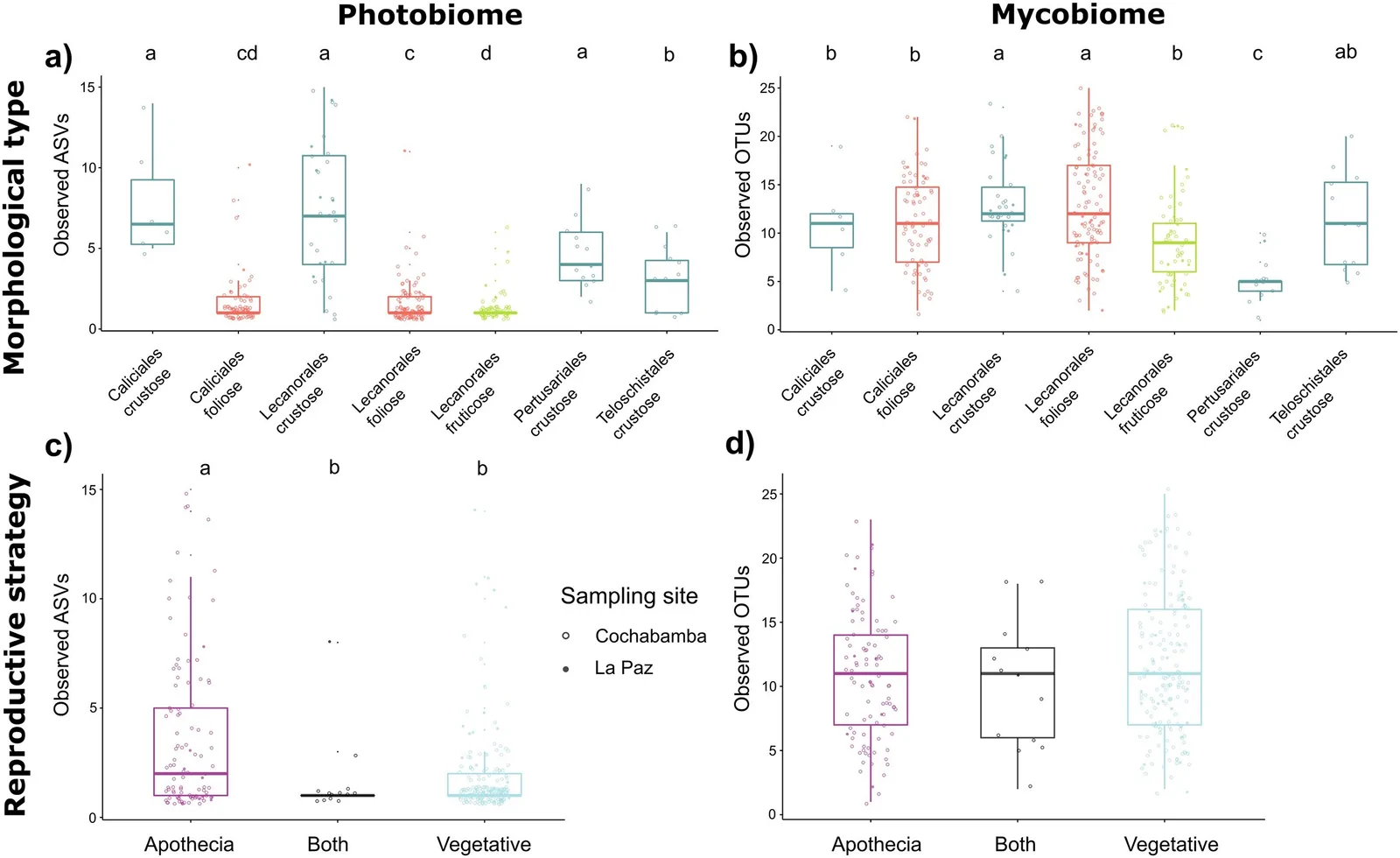
Alpha diversity of lichen-associated photobiome and mycobiome grouped by the combined effect of morphological type and mycobiont taxonomic assignation, and reproductive strategy. Observed richness, number of algal ASVs for the photobiome and fungal OTUs for the mycobiome, is shown grouped by explanatory factors: morphological type (a, b) and reproductive strategy (c, d). Significant differences between groups (*P* < 0.05) were determined using Kruskal–Wallis tests and are indicated by letters above the boxplots. For reproductive strategies, “apothecia” corresponds to lichens forming only apothecia; “vegetative” corresponds to lichens with one or two types of vegetative propagules (isidia or soredia); and “both” corresponds to lichens that produce apothecia and one or both types of vegetative propagules.

Thallus morphology significantly influenced both photobiome and mycobiome composition, although its effect was weaker than that of the mycobiont identity, which was confirmed by dbRDA and PERMANOVA analyses (Table S6a: thallus morphology explained 7.62% and 3.4% of the variation in photobiome and mycobiome composition, respectively, according to the dbRDA models; (Table S7a: PERMANOVA, R² = 0.08, *P* = 0.001 for the photobiome; R² = 0.03, *P* = 0.001 for the mycobiome). Thallus morphological type also significantly influenced alpha diversity, as measured by the richness of the photobiome (number of algal ASVs) and mycobiome (number of fungal OTUs) (Fig. 3a and b). Crustose lichens exhibited significantly higher algal richness (number of observed ASVs, Fig. 3a) and diversity (Shannon index, Fig. S5a) than foliose and fruticose forms. Fungal richness was significantly higher in crustose and foliose lichens than in fruticose representatives of Lecanorales, although this pattern varied among lichen orders (Fig. S5b).

#### Lichen reproductive strategy

Reproductive strategy had a statistically significant effect on photobiome community composition according to PERMANOVA (Table S7a: R² = 0.09, *P* = 0.001), although the explanatory power of this factor was low in the dbRDA framework, which attributed less than 1% of the total variance to reproductive strategy (Table S6a). This indicates that while compositional differences are detectable, they are subtle in magnitude.

Consistent with this, lichens forming only apothecia (sexual reproductive structures producing spores) hosted significantly higher photobiome diversity, including increased algal ASV richness (Fig. 3c, S6a) and higher Shannon diversity (Fig. S5c, S6c), compared to lichens producing isidia and/or soredia (asexual propagules). No significant differences in photobiome richness or Shannon diversity were detected between lichens with isidia and those with soredia (Fig. S6a, c).

In contrast, the mycobiome showed weaker and less consistent associations with reproductive strategy. PERMANOVA indicated a statistically significant but very small effect on fungal community composition (Table S7a), and dbRDA likewise explained less than 1% of variance (Table S6a). Neither fungal richness (observed OTUs) nor community composition differed significantly among reproductive strategies at the broader level (Fig. 3d, S6b, d). However, within asexually reproducing lichens, isidiate individuals showed higher fungal OTU abundance compared to sorediate individuals (Fig. S6b).

Overall, these results suggest that reproductive strategy has a detectable but weak structuring effect on lichen-associated microbial communities, with a clearer signal in the photobiont than in the mycobiome.

#### Environmental factors

Spatial variation between sampling sites had a limited effect on photobiome and mycobiome composition, as we observed minor and non-significant differences in community composition and alpha diversity (Fig. S7) between the two most (n = 261) sampled sites (CB1 and CB2). Between sites located in Cochabamba (CB) and La Paz (LP), we observed slight differences in photobiome community composition and algal richness (Fig. S7). However, the photobiomes and mycobiomes at all four sites shared fungal OTUs and algal ASVs, which indicates the retention of the same communities (CB3 was removed in this comparison, as in the final resolution, this site is represented by less than 10 samples). Rarefaction analyses indicated higher observed richness and phylogenetic diversity of photobiont and mycobiome taxa at the undisturbed site (CB2) compared to the more disturbed site (CB1; Fig. S1). In contrast, mycobiont observed richness showed substantial overlap between sites and was broadly comparable across sampling depths, whereas phylogenetic diversity was consistently higher in CB2 than in CB1 (Fig. S1). The sampling site had an overall negligible effect on community compositions of all sampling sites (Table S6: sampling site explained 4.15% and 3.59% of the variation in photobiome and mycobiome composition, respectively, according to the dbRDA models; Table S6a; R² < 0.05, *P* = 0.001).

#### Lichen secondary metabolites

We observed that lichens with similar secondary metabolite composition (Table S1) tended to host similar photobiome and mycobiome communities (Mantel tests; Table S8a). In particular, beta-orcinol depsides and terpenoids had the most significant effect on the photobiome and mycobiome communities in all lichen specimens in which these secondary metabolite groups were identified (Table S8b). In particular, beta-orcinol depsides and terpenoids were associated with a significantly larger proportion of saprotrophic and symbiotrophic fungi (Wilcoxon tests: *P* < 0.01). Conversely, orcinol depsides were associated with a significantly increased proportion of pathotropic fungi (Wilcoxon tests: *P* = 0.02). In terms of alpha diversity, lichens devoid of secondary metabolites displayed, on average, a more diverse photobiome (Wilcoxon tests: *P* = 0.03), while those lacking beta orcinol depsides were colonized by a more diverse mycobiome (Wilcoxon tests: *P* = 0.003). Lichens with orcinol depsides simultaneously hosted a richer photobiome and mycobiome (Wilcoxon tests: *P* < 0.05).

### Network structures and specialization

We observed that direct and indirect interactions within lichen holobionts resulted in contrasting network structures and varying levels of specialization, based on communities from the two study sites (CB1 and CB2; Figs. S8–S11).

#### Mycobiont-photobiome network

**Figure 4:**
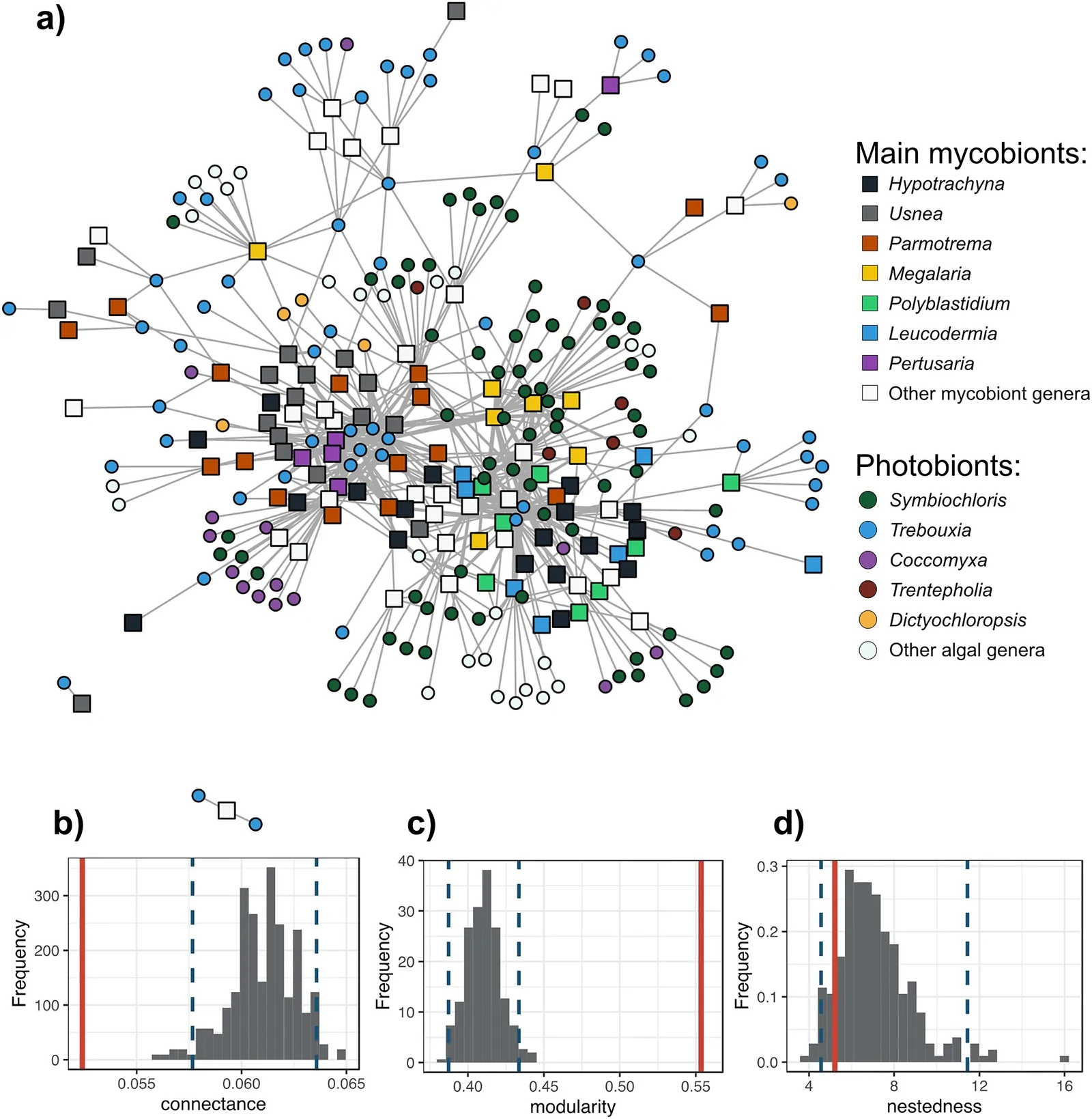
(a) Representation of the meta-network between all mycobionts and algae across the 309 lichens collected from five sampling sites. Each node represents a mycobiont OTU (square shape) or an algal ASV (circular shape), and each gray line indicates an interaction. The position of the nodes reflects the similarity in species interactions. OTUs and ASVs are colored according to their genus. (b–d) Evaluation of the structure of the mycobiont-algae network reconstructed in an undisturbed community of Cochabamba (CB2): we measured the original connectance (b), modularity (c), and nestedness (d) values (represented by vertical continuous lines) that we compared to the null expectations given by randomized networks (vertical dashed lines). Dashed lines delimit the estimated 95% confidence intervals.

The networks linking mycobionts and algae forming the photobiome showed low connectance (0.05; Fig. 4; Fig. S8a) and elevated specialization (H2′ > 0.20; Fig. S9) relative to null expectations. Nestedness was low in both CB1 (10) and CB2 (5), and did not differ significantly from randomized network expectations (Fig. S8a). Network-level specialization (H2′) was comparable between the disturbed site (CB2) and the undisturbed site (CB1; Fig. S9). Importantly, the algal OTU-based analyses yielded qualitatively identical results to those obtained using algal ASVs, including the relative differences between sites (Fig. S10). Furthermore, the analysis of the same parameters (connectance, nestedness, H2’) using algal OTUs demonstrates that our conclusions regarding network structure and specialization are robust to the choice of algal taxonomic resolution (Fig. S10).

At the level of growth forms, H2′ values were higher in foliose lichens compared to crustose and fruticose taxa, but not uniform across the disturbed (CB2) and the undisturbed site (Fig. S11a). Whereas differences among reproductive strategies were more variable, in particular, vegetatively reproducing lichens tended to show higher specialization than sexually reproducing taxa (Fig. S11b).

Significant phylogenetic signal was detected in mycobiont–interaction patterns, indicating that closely related fungal taxa tend to associate with more similar photobiont communities (Table S9). In contrast, phylogenetic signal in photobiont association patterns was weaker and depended on the distance metric used, with no consistent support across all analyses. These results are based on Mantel tests using Spearman correlations with 10 000 permutations. Importantly, this asymmetry should not be interpreted as a uniform “both directions” effect: phylogenetic structuring is robust for mycobionts but only conditionally supported for photobionts depending on the metric applied.

This pattern is further reflected in the meta-network structure, where taxa belonging to the same genus tend to cluster in interaction space, forming modular groups of closely connected nodes (Fig. 4a, S12a). Such clustering indicates that interaction partners are not randomly distributed across the phylogeny, but instead show taxonomic structuring consistent with the detected phylogenetic signal.

At the level of individual taxa, significant NRI values were detected for several mycobiont lineages, suggesting phylogenetic clustering of associated partners within these lineages, whereas algal lineages showed no consistent signal of phylogenetic structuring in their associations (Fig. S13a). Specialist mycobionts were particularly frequent within the genera *Megalaria* and *Pertusaria*.

#### Mycobiont–mycobiome network

Networks describing interactions between mycobionts and their associated mycobiome were modular with low nestedness, and exhibited a specialization based on both connectance and H2′ (Figs. S8b, S9). However, overall specialization (H2′ = 0.12–0.20) was lower than that observed in mycobiont–photobiome networks (H2′ > 0.20). Network-level specialization (H2′) varied between sampling sites, with higher values at the undisturbed site (CB2; Fig. S9), and showed only minor variation across functional traits (thallus morphology and reproductive strategy; Fig. S11a-b). Foliose lichens tended to exhibit lower levels of specialization (H2′ ≤ 0.20; Fig. S11a), whereas foliose and fruticose forms showed comparatively higher specialization at the disturbed site relative to the undisturbed site.

Phylogenetic signal in interaction patterns was weaker and less consistent in mycobiont–mycobiome associations than in mycobiont–photobiome networks, and varied depending on network component, site, and distance metric (Fig. S12b; Table S9). In particular, no significant signal was detected in CB1 under either Bray–Curtis or UniFrac distances (R = 0.07 and 0.03, respectively; *P* > 0.05). In contrast, CB2 showed a significant moderate phylogenetic signal under both metrics (Bray–Curtis: R = 0.28, *P* = 0.001; UniFrac: R = 0.20, *P* = 0.005), indicating that closely related taxa tend to associate with more similar partners in the disturbed site. This indicates that phylogenetic structuring of interactions is not consistent across environmental conditions, but becomes detectable under disturbed conditions (CB2), suggesting environment-dependent organization of interaction patterns.

At the level of individual taxa, evidence for specialization between mycobiont genera and mycobiome-forming fungal orders was generally limited (Fig. S13b), with the majority of genus–order associations (∼two-thirds) showing non-significant NRI values. However, patterns differed among ecological guilds within the mycobiome: symbiotrophic fungi exhibited higher levels of specialization (i.e. more positive and significant NRI values) compared with saprotrophic and pathotrophic fungi (Fig. S14).

#### Photobiome–mycobiome network

Similarly, interactions between the photobiome and mycobiome formed modular, low-nestedness networks (Fig. S8c). However, these networks exhibited significant, but relatively low levels of specialization (H2′ < 0.12; Fig. S9), and moderate phylogenetic signal (Table S9).

At the level of individual taxa, we detected intermediate levels of specialization between photobiome-forming algal species and mycobiome-associated fungal orders (Fig. S13c). The algal OTU-based analyses of mycobiome–photobiome networks produced broadly similar patterns to those obtained using algal ASVs, although the absolute values of several network metrics differed (Fig. S10). In particular, OTU-based networks exhibited higher connectance, lower modularity, and higher nestedness than the corresponding ASV-based networks, while H2′ values remained within a comparable range. Nevertheless, the relative differences between sampling sites were largely maintained (Fig. S10).

Significant phylogenetic signal was detected in mycobiome-interaction patterns, indicating that closely related non-lichenized fungal taxa tend to associate with more similar photobiome communities (Table S9). In contrast, phylogenetic signal in photobiome association patterns was weak and largely unsupported, with significant correlations detected only for one site (CB1) under a single distance metric, whereas no significant signal was found for the remaining analyses. Thus, phylogenetic structuring of interaction patterns is robust for the mycobiome but absent or only weakly supported for the photobiome.

## Discussion

Using a combination of functional traits and network-based analyses, we showed that lichen photobiome and mycobiome structure is primarily structured by mycobiont identity, with additional contributions from thallus morphology, reproductive strategy, and secondary metabolites.

### Lichen functional traits shape photobiome and mycobiome structure

Lichen thallus morphology reflects repeated evolutionary innovations (Spribille et al. 2022) and is linked to ecological function (Gauslaa 2014). In our dataset, crustose lichens consistently harbored a richer and more diverse photobiome than foliose and fruticose forms, even within the same lichen mycobiont order. This pattern is consistent with previous findings (Helms et al. 2001) and may be explained by the close contact of crustose thalli with the substrate on which lichens grow, facilitating the incorporation of environmental algae (Sanders 2014). In contrast, the more complex, corticated structure of foliose and fruticose lichens may restrict access to external photobionts. Although statistically significant, functional traits such as thallus morphology explained a relatively modest proportion of variation in photobiome and mycobiome composition (generally ∼3%–10%), indicating that they are consistent but partial predictors of community structure, with additional unexplained variation likely reflecting other ecological and stochastic factors.

For the mycobiome, differences between lichen morphological types were less consistent and appeared to depend on lichen taxonomic context. Within Lecanorales, fruticose and crustose species exhibited higher fungal diversity than foliose forms, suggesting that physical substrate contact may also facilitate fungal colonization. However, differences among mycobiont orders indicate that mycobiont identity and associated traits, including composition of secondary metabolites, likely override morphological effects. For example, lower fungal diversity in crustose Pertusariales may reflect the influence of specific chemical compounds such as xanthones and β-orcinol depsidones, which possibly inhibit the colonisation of lichen thalli by fungi. Furthermore, most fruticose representatives of Lecanorales included in this study produce usnic acid derivatives and β-orcinol depsidones, in contrast to foliose and crustose representatives, which present different chemical profiles. These compounds may influence the diversity and composition of the lichen-associated mycobiome, potentially confounding the apparent effect of thallus morphology. However, this interpretation should be treated with caution, as fruticose lichens were represented by only two genera, *Usnea* and *Ramalina*, with uneven sampling. Larger taxonomic coverage will be necessary to disentangle the relative contributions of morphology and secondary chemistry in shaping mycobiome structure. Overall, these results suggest that thallus morphology alone does not determine mycobiome structure but interacts with taxonomy-specific traits like secondary metabolite composition.

Reproductive strategy influenced photobiome diversity, primarily affecting the number of associated algal ASVs, but had limited effects on the mycobiome. Our results confirmed previously observed patterns that: (I) lichen fungi reproducing sexually *via* spores harbored a broader range of algal partners, consistent with horizontal acquisition from the environment (Wornik and Grube 2010, Cao et al. 2015, Steinová et al. 2019); whereas (II) vegetatively reproducing lichens showed lower photobiome diversity, reflecting constrained algae transmission. However, potential photobiont switching (Nelsen and Gargas 2008, 2009, Muggia et al. 2008, Magain and Sérusiaux 2014, Ohmura et al. 2019) may partially offset this constraint. Our study did not consider this phenomenon, which needs more attention in future studies.

In contrast, mycobiome diversity did not differ between reproductive modes, suggesting that fungal communities are largely acquired from the environment rather than vertically transmitted. Higher OTU abundance in isidiate lichens compared to sorediate ones may reflect structural differences, as isidia provide more protected microhabitats, whereas soredia are more exposed during dispersal. Nevertheless, evidence for vertical transmission of the mycobiome remains limited.

Secondary metabolites influenced both photobiome and mycobiome composition. Lichens lacking secondary metabolites identified by TLC harbored more diverse photobiomes, suggesting that the presence of these compounds may restrict certain algal lineages (Bačkor et al. 1998, 2010, Lokajová et al. 2014). However, the higher diversity observed in lichens containing orcinol depsides indicates that some photobionts may tolerate or adapt to these metabolites (Bačkor et al. 1998, 2010, Lokajová et al. 2014). In the case of lichens that produced β-orcinol depsides (atranorin) and terpenoids (zeorin), they were associated with increased proportions of saprotrophs and symbiotrophs within their mycobiome, whereas orcinol depsides (evernic and lecanoric acids) correlated with higher abundance of pathotrophs, indicating differential tolerance among fungal guilds. These findings support previous observations that lichen-associated fungi can adapt to host metabolites (Hawksworth 1982, Lawrey et al. 1999, Torzilli et al. 1999), although our results remain correlative.

Regardless, an important factor to consider in future studies is the localization of secondary metabolites within lichen thalli, to determine whether algal cells are in direct contact with these compounds, as well as their concentrations. Moreover, quantifying specific compounds in relation to environmental factors could provide insight into lichen responses to environmental change, including potential parasitic fungal infections. Furthermore, lichen secondary metabolites are linked to the genetics of the mycobiont (Singh 2023); therefore, their potential effects on photobiome and mycobiome composition may be associated with mycobiont identity. Lichen model species exhibiting multiple chemotypes may thus be ideal systems for investigating how secondary metabolites influence the structure of lichen holobionts.

From the perspective of the holobiont concept, our results are broadly consistent with patterns described in other hosts, namely plants, where host identity (plant species) is also a primary determinant of microbiome assembly (Mesny et al. 2023, Rodríguez et al. 2025). Likewise, in plants, phenotypic traits, such as for example root architecture (e.g. Edwards et al. 2015), leaf morphology (e.g. Zhai et al. 2025), and secondary chemistry (e.g. Bressan et al. 2009), which are dynamic factors, influence microbial colonization and community composition, over plant growth, although their effects are secondary compared to plant host identity. Notably, the role of secondary metabolites as selective agents is consistent across both systems, in which plant root exudates and lichen compounds similarly act as chemical filters that influence microbial diversity and functional composition.

### Network structure reveals asymmetric specialization

Network analyses showed that interactions among studied holobiont components are modular and non-nested, with low connectance—features typical of intimate symbiotic systems (Guimarães Jr et al. 2007, Fontaine et al. 2011). However, levels of specialization differed markedly among interaction types.

The mycobiont–photobiome network exhibited high specialization and strong phylogenetic signal, indicating that closely related fungi associate with similar photobionts and vice versa. This pattern supports a tightly constrained and potentially co-evolved relationship. In contrast, networks involving the mycobiome showed lower specialization and weaker phylogenetic structure, suggesting more flexible and environmentally influenced associations. These results challenge the assumption of strong co-speciation between lichens and their associated fungi, especially lichenicolous fungi (Werth et al. 2013, Diederich et al. 2018), and instead support a model in which the mycobiome is only partially structured by host mycobiont identity. The observed influence of the mycobiont identity on symbiotrophic and pathotrophic fungi, but not saprotrophs, further supports the idea that some components of the mycobiome are host-filtered, whereas others are environmentally derived.

The relatively low but significant level of interaction between the photobiome and mycobiome suggests limited direct coupling between these communities. Nevertheless, the presence of phylogenetic signals across all networks indicates that holobiont structure may influence the joint evolution of its components. In contrast, plant–microbe interactions are generally more flexible and less specific, particularly for bacteria and fungi in the rhizosphere (Bulgarelli et al. 2013, Bergelson et al. 2019). This difference likely reflects the obligate nature of the lichen symbiosis, in contrast to plants, which can recruit diverse and interacting microbial communities from the environment, collectively referred to as the plant microbiota (Hassani et al. 2018).

### Effects of anthropogenic disturbance

Although studied environmental factors (locality, habitat type, or anthropogenic activity) had a limited effect on overall microbial community composition, the structure of the interaction network revealed sensitivity of lichen holobiont components to environmental disturbance. In our study, anthropogenic disturbance was associated with habitat fragmentation due to grazing or logging, which can alter microclimatic conditions and reduce substrate continuity, thereby limiting the dispersal and establishment of both photobionts and associated fungi. In line with this, specialization in mycobiont–photobiont and mycobiont–mycobiome interactions tended to decrease under disturbed conditions, particularly in foliose and fruticose lichens. This pattern is consistent with previous studies showing reduced specialization under habitat fragmentation (Berlinches de Gea et al. 2023, Blázquez et al. 2025). Crustose lichens appeared less affected, possibly due to their ability to incorporate environmental micro-organisms during growth. Overall, reduced specialization under disturbance may reflect a shift toward more generalist interactions, potentially weakening symbiotic stability and resilience. The response to environmental disturbance shows convergence across lichen and plant holobionts. In both lichens (Berlinches de Gea et al. 2023, Blázquez et al. 2025) and plants (Xiao et al. 2016), disturbance tends to reduce interaction specialization and promote more generalist associations, likely reflecting a breakdown of stable symbiotic relationships and increased influence of environmental recruitment. This shift may have implications for holobiont stability and resilience under changing environmental conditions.

### Perspectives

Our conclusions are constrained by limitations in taxonomic and functional annotation of the mycobiome, particularly the reliance on FUNGuild and the high proportion of unassigned OTUs. In addition, we adopted a community ecology approach to characterize lichen-associated microbial assemblages under natural field conditions. However, sampling was uneven across taxa due to their natural occurrence and varying abundances in the studied habitats, which may introduce biases in estimates of diversity and network structure. Although this reflects ecological realism, it limits equal representation of all host groups and should be considered when interpreting comparative patterns.

Future studies should integrate bacterial and viral components of the lichen holobiont, which remain understudied but may play important roles in community structure and function (Aschenbrenner et al. 2014, Ponsero et al. 2021). Combining multi-omics approaches with experimental validation will be essential to disentangle the mechanisms underlying holobiont assembly.

## Conclusion

Overall, our results indicate that within the lichen holobiont, the mycobiont identity is the primary determinant of both photobiome and mycobiome assembly, while lichen specimens’ functional traits and the environmental conditions of their habitat act as secondary drivers. Notably, crustose lichens harbored significantly higher algal richness and diversity than other growth forms, suggesting that lichen functional traits can influence the availability and maintenance of photobiont diversity within the holobiont. Network analyses, performed to disentangle biotic interactions between the different microbial components within the lichen holobiont, reveal asymmetric specialization, with strong phylogenetic structuring in mycobiont–photobiont interactions but weaker, more flexible associations involving the mycobiome. This contrast suggests that photobiont associations are more tightly constrained and potentially co-evolved, whereas the mycobiome is more loosely structured and likely influenced by environmental recruitment. These findings highlight the importance of host-driven filtering and interaction asymmetry in shaping lichen holobionts and provide a framework for understanding their responses to environmental change.

## Supplementary Material

fiag094_Supplemental_Files

## Data Availability

Short-read data generated in this study were deposited in the NCBI Sequence Read Archive with BioProject accession no. PRJNA1117370, which includes fungal and algal ITS2 rDNA sequencing data under BioSample accessions SAMN41564377 and SAMN41564378.
