# Supplementary material for: Factors shaping the lichen photobiome and mycobiome in a tropical lichen community
Source: FEMS Microbiol Ecol. 2026 Aug 14;102(9):fiag094. doi: 10.1093/femsec/fiag094 (PMC13508176; doi:10.1093/femsec/fiag094)
Supplement: Supplementary file 1 [file fiag094_supplemental_files.zip › Supplementary_data_29_06_2026.docx]

## **Supporting Information**

**Article title:** **Factors shaping the lichen photobiome and mycobiome in a tropical lichen community**

**Authors**: Magdalena Kosecka, Amélia Bourceret, Benoît Perez-Lamarque, Beata Guzow-Krzemińska, Martin Kukwa, Adam Flakus, Pamela Rodriguez-Flakus, Marc-André Selosse

**The following Supporting Information is available for this article:**

**Supplementary Figures:**

**Fig. S1:** Rarefaction curves for mycobiont, photobiome, and mycobiome diversity across sampling sites.

**Fig. S2:** Photobiome and mycobiome composition across lichen specimens at the genus level.

**Fig. S3:** Photobiome composition across lichen specimens based on algal OTUs.

**Fig. S4:** The relative abundances of the trophic mode of lichen-associated mycobiome grouped by mycobiont taxonomic assignment at the order and the genus level.

**Fig. S5:** Alpha diversity of lichen-associated photobiome and mycobiome, grouped by the combined effect of morphological type and mycobiont taxonomic assignation, and reproductive strategy.

**Fig. S6:** Alpha diversity (observed richness and Shannon index) of lichen-associated photobiome and mycobiome, grouped by the lichen reproductive strategy.

**Fig. S7:** Relative abundance and alpha diversity of the most abundant mycobionts (a), algae (b), and mycobiome taxa, across the sampling sites.

**Fig. S8:** The results of connectance, modularity, and nestedness measured for bipartite interaction networks.

**Fig. S9:** The results of the network-level H2’ index of interaction specialization.

**Fig. S10:** Results of connectance, modularity, nestedness, and H2′ calculated for mycobiont–photobiome and photobiome–mycobiome bipartite interaction networks using algal OTUs instead of ASVs.

**Fig. S11:** The results of the network-level H2’ index of interaction specialization for groups of data according to the lichen morphological type, reproduction strategy, and site conditions.

**Fig. S12:** Visualization of the different types of networks: mycobiont–photobiome, mycobiont–mycobiome, and photobiome–mycobiome.

**Fig. S13:** Results of the Net Relatedness Index (NRI) by holobiont component identity.

**Fig. S14:** Results of the Net Relatedness Index (NRI) according to mycobiome ecological guilds inferred by FUNGuild.

**Supplementary Materials:**

**Method S1:** Lichen-forming fungi molecular identification by Sanger sequencing.

**Method S2:** Amplicon sequences data processing pipeline and script.

**Supplementary Figures:**

## **Fig. S1: Rarefaction curves for mycobiont, photobiome, and mycobiome diversity across sampling sites.**

Rarefaction curves indicate that communities sampled in La Paz (LP1 and LP2) were far from reaching saturation, whereas saturation was approached more closely in Cochabamba (CB1 and CB2). Within Cochabamba, diversity was consistently higher in the undisturbed site (CB2) than in the disturbed site (CB1).

For the mycobiont and mycobiome datasets, rarefaction curves show the expected number of fungal OTUs (Richness) and phylogenetic diversity (Faith’s PD) as a function of the number of sampled lichen specimens. For the photobiome, rarefaction curves show the expected number of algal ASVs and OTUs, demonstrating that analyses based on ASVs and OTUs yielded comparable patterns.

## **Fig. S2:** **Photobiome (ASV) and mycobiome composition across lichen specimens at the genus level.**

PCoA based on Bray-Curtis dissimilarity obtained from all specimens (n=309). Shapes indicate the sample site (Cochabamba or La Paz), whereas colors present the mycobiont identity at the genus level (a, c) (n=309). The shaded polygons (clouds) delineate mycobiont identities at the genus level. The dbRDA and PERMANOVA analyses revealed significant differences within mycobiont genera in both photobiome and mycobiome (dbRDA explained 25.46% and 14.71% of the variation, respectively, p = 0.001; Table S5: PERMANOVA: R^2^=0.26; 0.15, respectively, p = 0.001). Relative abundances of the most abundant algal (b) and fungal (d) groups at the species and order levels, respectively. The main orders that represent most of the reads are included, while some other taxa, that represent the rest of the reads to reach 100%, are not shown. The legend of relative abundances of the most abundant algae (b) is organized by alphabetical order, and in the case of fungi (d), the legend is ordered from the most abundant to the least abundant fungal orders.

## **Fig. S3: Photobiome composition across lichen specimens based on algal OTUs.**

This figure mirrors the ASV-based photobiome analyses presented in Fig. 2a-b and Figure S2a-b; it demonstrates that clustering patterns, community composition, and the effects of mycobiont identity are qualitatively consistent regardless of whether algal diversity is analysed at the ASV or OTU level.

Principal Coordinates Analysis (PCoA) based on Bray–Curtis dissimilarity was performed for all specimens at the order level (a), and genus level (c). Relative abundances of the most abundant algal OTUs are shown in b and d. The dbRDA and PERMANOVA analyses revealed significant differences among mycobiont orders and genera, consistent with the ASV-based analyses.

## **Fig. S4: The relative abundances of the trophic mode of lichen-associated mycobiome grouped by mycobiont taxonomic assignment at the order (a) and the genus (b) level.**

Relative abundances (RAs) of the trophic mode defined by using FunGuild database, in the total fungal community. After the verification, e.g., removing animal or plant pathotrophs.

## **Fig. S5: Alpha diversity of lichen-associated photobiome and mycobiome within individual lichen thalli, grouped by the combined effect of morphological type and mycobiont taxonomic assignation, and reproductive strategy.**

Alpha-diversity (Shannon index) of samples depends on the combined effect of morphological type and mycobiont taxonomic assignation (a, b) and on lichen reproductive strategies (c, d). A Kruskal-Wallis test (p<0.05) was used for statistical analysis. Letters indicate significant differences between conditions.

## **Fig. S6: Alpha diversity (observed richness and Shannon index) of lichen-associated photobiome and mycobiome within individual lichen thalli, grouped by the lichen reproductive strategy.**

Alpha-diversity (observed richness and Shannon index of algal ASVs and fungal OTUs) of samples depending on reproductive strategy (A: apothecia, AVI: apothecia and isidia, AVIVS: apothecia, isidia and soredia, AVS: apothecia and soredia, ST: sterile, VI: isidia, VIVS: isidia and soredia, VS: soredia). A Kruskal-Wallis test (p<0.05) was used for statistical analysis. Letters indicate significant differences between conditions.

## **Fig. S7: Relative abundance and alpha diversity of the most abundant (a) algae and (b) mycobiome taxa, across** **the sampling sites.**

Relative abundances (RAs) of the most abundant algae (a) and mycobiome taxa (b) are shown at the species, and order levels, respectively; alpha diversity, measured as observed richness (algal ASVs and fungal OTUs; c, d) and Shannon index (e, f), presented across sampling localities (LP1, LP2, CB1, and CB2). CB3 was excluded from the analysis due to a low sample size (< 10 samples). Statistical differences among localities were assessed using Kruskal–Wallis tests (p < 0.05), and significant differences are indicated by letters.

## **Fig. S8: The results of connectance, modularity, and nestedness measured for bipartite interaction networks.**

For each site (CB1 and CB2) and each type of network: (a) mycobiont–photobiome, (b) mycobiont–mycobiome, and (c) photobiome–mycobiome, we measured the original connectance, modularity, and nestedness values (represented in red) that we compared to the null expectations given by randomized networks (in gray). Dashed lines delimit the estimated 95% confidence intervals.

## **Fig. S9: The results of the network-level H2’ index of interaction specialization.**

For each site and each type of network, we measured the original H2’ value (represented in red) that we compared to null expectations given by randomized networks (in gray). Dashed lines delimit the estimated 95% confidence intervals. An H2’ value close to 0 indicates low specialization, whereas H2’ value close to 1 indicates high specialization.

## **Fig. S10: Results of connectance, modularity, nestedness, and H2′ calculated for mycobiont–photobiome and photobiome–mycobiome bipartite interaction networks using algal OTUs instead of ASVs.**

For each site (CB1 and CB2) and (a) mycobiont–photobiome, and (b) photobiome-mycobiome network, we measured the original connectance, modularity, nestedness and H2’ index values (represented in red) that we compared to the null expectations given by randomized networks (in gray). Dashed lines delimit the estimated 95% confidence intervals.

## **Fig. S11: The results of the network-level H2’ index of interaction specialization for groups of data according to the lichen morphological type, reproduction strategy, and site conditions.**

The analysis was conducted for bipartite networks of mycobiont–photobiome, mycobiont–mycobiome, and photobiome–mycobiome grouped by crustose, foliose and fruticose lichen morphological type separately (a), and for the same networks, but grouping according to the reproduction strategy - “apothecia” corresponds to lichens forming only apothecia; “vegetative” corresponds to lichens with one or two types of vegetative propagules (isidia or soredia); and “both” corresponds to lichens that produce apothecia and one or both types of vegetative propagules.

The H2’ index indicates that the strength of mycobiont-photobiome specialization is higher in foliose lichens with (at least partially) vegetative reproduction.

**photobiome-mycobiome, vegetative**

**photobiome-mycobiome, apothecia**

**mycobiont-photobiome, vegetative**

**mycobiont-mycobiome, both**

**mycobiont-mycobiome, apothecia**

**mycobiont-mycobiome, vegetative**

**mycobiont-photobiome, both**

**mycobiont-photobiome, apothecia**

**photobiome-mycobiome, both**

## **Fig. S12: Visualization of the different types of networks: mycobiont–photobiome, mycobiont–mycobiome, and photobiome–mycobiome.**

Each node represents an OTU (either a mycobiont, or the mycobiome) or ASV (the photobiome), and each gray line indicates that there exists an interaction between two OTUs/ASVs, and their widths are proportional to the number of times the interaction has been observed. The position of the nodes reflects the similarity in species interactions.

In the case of comparison between disturbed (CB1) and undisturbed (CB2) communities, the shapes are slightly different, especially for the network of mycobionts and the mycobiome (b), which reflects a decrease in specialization level.

**Fig. S13: Results of the Net Relatedness Index (NRI) by holobiont component identity.**

For each site (CB1 and CB2), we measured, in each network type (a) mycobiont-photobiome, (b) the mycobiont-mycobiome, or (c) the photobiome-mycobiome, the NRI values for each OTU/ASV and assessed for significance using null-model permutations. Here, only the NRI values are plotted; significance (derived from permutation p-values) is indicated by color (green = significant, purple = non-significant).

We tested the effect of the taxonomic groups on the NRI values using a Kruskal-Wallis test (reported above each panel).

The presence of two values for certain taxonomic labels (e.g., mycobiont genera such as *Usnea*, *Parmotrema*, *Hypotrachyna*, as well as some algal species and mycobiome-forming fungal orders) arises because multiple distinct OTUs/ASVs were assigned to the same higher-level taxonomic category. Each OTU/ASV retains its independently calculated NRI value and associated significance; values were not merged or collapsed based on significance.

(a) **mycobiont-photobiome network**

**Mycobionts**

**Photobiome**

**(b) mycobiont-mycobiome network**

**Mycobiome**

**Mycobionts**

**(c) photobiome-mycobiome network**

**Mycobiome**

**Photobiome**

## **Fig. S14: Results of the Net Relatedness Index (NRI) according to mycobiome ecological guilds inferred by FUNGuild.**

For each site (CB1 and CB2), NRI values were calculated at the OTU/ASV level for the mycobiont–mycobiome and photobiont–mycobiome networks, and statistical significance was assessed using permutation tests. Each OTU/ASV was then assigned to an ecological guild (pathotroph, symbiotroph, or saprotroph) using FUNGuild. NRI values are plotted as individual OTU/ASV-level observations, grouped by assigned guild for visualization. Significant positive NRI values are indicated in green, and non-significant values in purple; no aggregation or filtering of OTU/ASV values was performed based on significance.

We tested the effect of the taxonomic groups on the NRI values using a Kruskal-Wallis test (reported above each panel).

NRI results indicate that levels of specializations of mycobiome tend to be higher for symbiotrophs compared with saprotrophs or pathotrophs.

# **Supplementary Methods**

## **Method S1: Lichen-forming fungi molecular identification by Sanger sequencing.**

Genomic DNA was used for PCR amplifications of the internal transcribed spacer region (nucITS) using ITS1F (Gardes & Bruns 1993) and ITS4 primers (White et al. 1990). The same primers were used for sequencing. StartWarm HS-PCR Mix (A&A Biotechnology) was used for amplification. The following conditions were used for amplification of the ITS rDNA marker: initial denaturation at 95 ºC for 5 min, followed by 35 cycles at 95 ºC for 40 s, 54 ºC for 45 s and 72 ºC for 1 min, with a final elongation step at 72 ºC for 10 min. PCR products were visualized on agarose gels in order to determine DNA fragment lengths. Subsequently, PCR products were purified using Wizard SV Gel and PCR Clean-Up System (Promega). Sequencing was performed in Macrogen (the Netherlands/South Korea).

The newly generated nucITS rDNA sequences were compared with sequences available in the GenBank database (http://www.ncbi.nlm.nih.gov/BLAST/) using the Megablast search (Altschul et al. 1990) and used as additional species verification, presented in the main text in the section ‘Taxonomic Identification and Functional Trait Assessment’, and further mycobiont OTUs verification, presented in the main text in the section ‘Amplicon sequencing data processing’. The newly obtained sequences were deposited in GenBank with specific accession numbers (Table S1.1).

Altschul SF, Gish W, Miller W, Myers EW, Lipman DJ. Basic local alignment search tool. J Mol Biol. 1990 Oct 5;215(3):403-10. doi: 10.1016/S0022-2836(05)80360-2.

Gardes M, Bruns TD. ITS primers with enhanced specificity for basidiomycetes--application to the identification of mycorrhizae and rusts. Mol Ecol. 1993 Apr;2(2):113-8. doi: 10.1111/j.1365-294x.1993.tb00005.x.

White TJ, Bruns T, Lee S, et al. Amplification and direct sequencing of fungal ribosomal RNA genes for phylogenetics. In: Innis N, Gelfand D, Sninsky J and White T (eds). *PCR Protocols: A Guide to Methods and Applications*. San Diego: Academic Press, 1990, 315–322.

## **Method S2: Amplicon sequences data processing pipeline and script.**

Amplicon sequencing outputs from the two libraries (the ‘fungal dataset’ and ‘algal dataset’) were separately processed, following the scheme:

1. Merging:

- check quality encoding
- merging forward and reverse sequences (R1 and R2)
- check quality of merged sequences

2. Demultiplexing:
   - checking mapping file
   - remove bad quality sequences
   - reverse complement merged sequences
   - demultiplexing, create one file per sample
   - dereplicate sequences at the study level
   - cut primers and barcode
3. Database preparation

- download Data Base for a specific region
- cut Data Base with our primers

4. Clustering

- merge all sequences from all sample
- dereplicate, sort by size (abundance)
- clustering at 97% by using Data Base
- reorganization table
- make stats
- chimera identification
- taxonomic assignment of OTUs/ASVs
- Prepare raw OTU/ASV table
- OTU/ASV table filtration with negative controls

Below we provide the description of the files provided from the study, available at the following link: <https://osf.io/cn9gt/overview?view_only=f04f2dcad79345eb8dbf495b2135b68d>:

1. A pipeline and script for filtering of negative controls for the ‘fungal dataset’:

- Fungi reads Metabarcoding Pipeline for OTU
- Cleaning_fungi_table_negcontrol

2. A pipeline for OTUs and ASVs clustering, and a script for filtering of negative controls for the ‘algal dataset’:

- Algal reads Metabarcoding Pipeline for OTU and ASV
- Cleaning_algal_table_negcontrol

3. The output OTU/ASV tables were as follows:

**‘fungal dataset’:**

(i) **all the lichenized fungi OTUs:**
    a. OTU_table_ITSf_sorted_mycobionts
    b. OTU_table_ITSf_sorted_mycobionts_count
    c. OTU_table_ITSf_sorted_mycobionts_taxonomy
    d. table_main_mycobiont_per_sample

(filtered final list of mycobiont OTUs used for further analysis)

(ii) **all mycobiome OTUs:**
     a. OTU_table_ITSf_sorted_other_fungi
     b. OTU_table_ITSf_sorted_other_fungi_count
     c. OTU_table_ITSf_sorted_other_fungi_taxonomy
     d. OTU_table_ITSf_sorted_other_fungi_FUNGuild
     e. OTU_table_ITSf_sorted_other_fungi_symbiotroph
     f. OTU_table_ITSf_sorted_other_fungi_pathotroph
     g. OTU_table_ITSf_sorted_other_fungi_saprotroph

**‘algal dataset’:**

(iii) **all algal ASVs:**
      a. ASV_table_ITSa_sorted_algae_ASV
      b. ASV_table_ITSa_sorted_algae_ASV_count
      c. ASV_table_ITSa_sorted_algae_ASV_taxonomy
(iv) **all algal OTUs:**
     a. OTU_table_ITSa_sorted_algae_OTU97
     b. OTU_table_ITSa_sorted_algae_OTU97_count
     c. OTU_table_ITSa_sorted_algae_OTU97_taxonomy
4. The pipeline and script for the network and network-associated parameters analysis:
   a. script_network_lichens
